# Retraction: Lead induces the upregulation of protein arginine methyltransferase 5 possibly by its promoter demethylation

**DOI:** 10.1042/BCJ20180167_RET

**Published:** 2025-04-01

**Keywords:** DNMTs, epigenetics, lead, MEP50, PRMT5, PRMT5 promoter

This article by Sukmana and Yang (2018), titled “The type IV pilus assembly motor PilB is a robust hexameric ATPase with complex kinetics”, published in the Biochemical Journal 475 (11): 1979–1993, DOI: 10.1042/BCJ20180167, is being retracted at the request of the Editorial Board and the corresponding author Zhaomin Yang. In 2022, Zhaomin Yang, along with Virginia Tech, contacted the journal to request a correction or retraction of Figure 7 due to an investigation that revealed discrepancies in the underlying data, making the results in this figure irreproducible. Zhaomin Yang agrees to the retraction but maintains the reproducibility and validity of results outside Figure 7. Andreas Sukmana was contacted but did not respond.

